# Oxidative Damage to the Salivary Glands of Rats with Streptozotocin-Induced Diabetes-Temporal Study: Oxidative Stress and Diabetic Salivary Glands

**DOI:** 10.1155/2016/4583742

**Published:** 2016-07-13

**Authors:** M. Knaś, M. Maciejczyk, I. Daniszewska, A. Klimiuk, J. Matczuk, U. Kołodziej, D. Waszkiel, J. R. Ładny, M. Żendzian-Piotrowska, A. Zalewska

**Affiliations:** ^1^Department of Health Care, Higher Vocational School, Noniewicza 10 Street, 16-400 Suwalki, Poland; ^2^Students' Scientific Group “Stomatological Biochemistry”, Department of Conservative Dentistry, Medical University of Bialystok, Sklodowskiej M.C. 24a Street, 15-274 Bialystok, Poland; ^3^Specialist Dental Practice, I. Daniszewska, Żeromskiego 5 Street, 15-225 Bialystok, Poland; ^4^Department of Conservative Dentistry, Medical University Bialystok, Sklodowskiej M.C. 24a Street, 15-274 Bialystok, Poland; ^5^County Veterinary Inspection, Zwycięstwa 26B Street, 15-959 Bialystok, Poland; ^6^Department of Emergency Medicine and Disaster, Medical University of Bialystok, Szpitalna 37 Street, 15-295 Bialystok, Poland; ^7^Department of Hygiene, Epidemiology and Ergonomics, Medical University of Bialystok, Mickiewicza 2c Street, 15-222 Bialystok, Poland

## Abstract

*Objective.* This study evaluated oxidative damage caused to the salivary glands in streptozotocin-induced diabetes (DM).* Materials and Methods.* Rats were divided into 4 groups: groups 1 and 2, control rats, and groups 3 and 4, DM rats. 8-Hydroxy-2′-deoxyguanosine (8-OHdG), protein carbonyl (PC), 4-hydroxynonenal protein adduct (4-HNE), oxidized and/or MDA-modified LDL-cholesterol (oxy-LDL/MDA), 8-isoprostanes (8-isoP), and oxidative stress index (OSI) were measured at 7 (groups 1 and 3) and 14 (groups 2 and 4) days of experiment.* Results.* The unstimulated salivary flow in DM rats was reduced in the 2nd week, while the stimulated flow was decreased throughout the duration of the experiment versus control. OSI was elevated in both diabetic glands in the 1st and 2nd week, whereas 8-isoP and 8-OHdG were higher only in the parotid gland in the second week. PC and 4-HNE were increased in the 1st and 2nd week, whereas oxy-LDL/MDA was increased in the 2nd week in the diabetic parotid glands.* Conclusions.* Diabetes induces oxidative damage of the salivary glands, which seems to be caused by processes taking place in the salivary glands, independently of general oxidative stress. The parotid glands are more vulnerable to oxidative damage in these conditions.

## 1. Introduction

Long-term metabolic disorders in the course of type 1 diabetes mellitus cause irreversible damage to many organs [1, 2]. The leading cause of diabetic complications is chronic hyperglycaemia, which induces a series of mechanisms that generate excessive production of reactive oxygen species (ROS). Diabetes is also associated with the reduction in the total antioxidant capacity, which results from disruption of endogenous antioxidant enzyme activities as well as increased formation of ROS. Events lead to the development of chronic oxidative stress in aerobic organisms [3].

The oxidative stress is defined as “a situation where the steady-state of ROS is transiently or chronically enhanced, disturbing cellular metabolism and its regulation and damaging cellular macromolecules” [4]. The target of ROS damage includes all groups of biomolecules which in the case of weakening of antioxidant systems may result in permanent changes in the redox state of DNA, RNA, proteins, lipids, and carbohydrates and leads to the loss of the biological function of the cells. It has been shown that the oxidation of amino acid residues results in a change of their native structure and biological activity of cellular proteins [5]. It has also been demonstrated that lipid peroxidation inhibits activity of enzymes, transporters, and receptors present in cell membranes [6]. The impact of ROS on DNA leads to damage of single nucleotide bases modifications, DNA double-strand breaks (DSBs), and formation of DNA adducts. The numerous oxidation markers are used to assess oxidative changes. The most widely used and applied approaches are generation of isoprostanes (8-isoP), oxidized and/or MDA-modified LDL-cholesterol (oxy-LDL/MDA), and reactive aldehydes, such as malondialdehyde (MDA) and 4-hydroxynonenal protein adduct (4-HNE protein adduct) [4], for protein-formation of protein carbonyls (PC) [7] for DNA 8-hydroxy-D-guanosine (8-OHdG) [8].

The concentration of reactive aldehydes such as malondialdehyde (MDA) is determined via their direct reaction with thiobarbituric acid (TBA). Direct TBA method is a nonspecific for MDA analysis. The pretreatment of biological samples for avoiding the possible interferences derived other reactive aldehydes which could be performed according to the recent sample preparation method [9]. It was shown that long-lasting streptozotocin-induced diabetes increased the MDA level only in the submandibular glands of rats in comparison to the control [10]. Deconte et al. [11] showed an increase in the MDA content in parotid glands of rats 30 days after STZ injection. Zalewska et al. [12] claimed that changes in MDA depend on the duration of streptozotocin-induced diabetes and the type of the salivary glands of rats.

The aforementioned oxidation biomarkers are among the most commonly used biomarkers, albeit not the only ones for the quantification of oxidative stress. Moreover, in certain cases, the levels of ROS modified molecules may also be decreased or unchanged as compared to the control due to their elimination by specific biological systems [4], which may falsely suggest a normal redox homeostasis. The oxidative stress index (OSI) is also used to fully illustrate the activity of oxidative stress. The OSI helps to assess the relationship between total oxidants (TOS) and total antioxidants (TAS) and may be regarded as a validated standard when assessing the oxidative stress [13].

The aim of our study was to assess the level and progression of oxidative damage measured by PC, 8-isoP, 4-HNE protein adduct, oxy-LDL/MDA, 8-OHdG, and OSI in the salivary glands of rats in two time periods of streptozotocin-induced diabetes.

The association between various oxidative stress markers such as PC, 8-isoP, 4-HNE protein adduct, oxy-LDL/MDA, 8-OHdG, and OSI and the secretory function of the salivary glands in the course of diabetes has not been studied so far. We aim to increase the understanding of the connection between oxidative stress and the dysfunction of salivary glands in two time periods of streptozotocin-induced diabetes.

## 2. Materials and Methods

### 2.1. Experimental Animals

Experiments were carried out in accordance with the European Communities Council Directive of 24 November 1986 (86/609/EEC) and in accordance with local laws and regulations. The protocol of the present study was approved by the Local Committee on the Ethical Use of Animals of the Medical University in Bialystok, Poland (resolution number 96/2015; date of approval 09.06.2015).

The experiment was carried out on male Wistar rats, caged separately, kept in standard animal holding conditions (at 21-22°C, in a cycle 12 h light/12 h dark, at constant humidity). Rats had unrestricted access to water and food (Agropol Motycz, Poland; consisting of 10.3% fat, 24.2% protein, and 65.5% carbohydrate). One week after arrival, the 8-week-old rats were divided randomly into control (C, *n* = 16) and diabetic (DM, *n* = 16), followed by a subdivision into two subgroups (8 rats in each subgroup) based on the number of days counted from the date of induction of diabetes in the DM group to the date of the end of the experiment (7 and 14 days).

### 2.2. Study Description

After overnight fasting, rats assigned to the DM groups were injected with a single, intraperitoneal dose of streptozotocin (freshly prepared in ice-cold citrate buffer) (STZ, Sigma Chemical Co., St. Louis, MO, USA; 50 mg/kg of body weight, sufficient diabetogenic effect, 100% the survival of rats) by the same experienced person [2, 12]. The control animals only received a citrate buffer (intraperitoneal injection). We chose the intraperitoneal method for simplicity of handling; moreover it was shown that intravenous injection of STZ caused mortality around 25% of the rats within the 1st week [14]. 48 hours after the STZ injection, the development of diabetes was confirmed by tail blood glucose analysis. Blood glucose levels, not urine glucose, were determined due to simplicity of handling. All 16 rats in both DM subgroups reached blood glucose level > 250 mg/dL, so they were considered diabetic. Seven and fourteen days after the STZ injection diabetic and control rats were anaesthetized by intraperitoneal injection with pentobarbital (80 mg/kg body weight), always in the morning (8.00–11.00) [12]. The rats were placed on a heated couch (37°C) at an angle 30°. Under anaesthesia, nonstimulated saliva secretion rate was measured for 15 min, using preweighted cotton balls inserted into the oral cavity [15]. Afterwards, salivation was stimulated with an intraperitoneal injection of pilocarpine nitrate (5 mg/kg BW, Sigma Chemical Co., St. Louis, MO, USA). Stimulated saliva secretion was measured in a way analogous to the unstimulated secretion, 5 minutes after the injection of pilocarpine, for 5 minutes [16]. The salivary flow rate was calculated by subtracting the initial weight from the final weight of the cotton balls. We assumed that 1 mg is equal to 1 *µ*L [17].

Next, still under anaesthesia, tail blood glucose analysis was performed (Accu-Check, Roche) followed by blood sampled from the abdominal aorta and the parotid and submandibular glands excised for further analysis. The salivary glands were weighted and immediately freeze-clamped with aluminium tongs, precooled in liquid nitrogen, and stored at −80°C. Blood was collected into glass tubes containing heparin and centrifuged (5 min, 4°C, 3000 g, MPW 351, MPW Med. Instruments, Warsaw, Poland). The obtained plasma was precooled in liquid nitrogen and stored at −80°C. No haemolysis was observed in any of the resulting plasma. The salivary glands and plasma were defrosted (4°C), washed with cold PBS, and reweighed. The salivary glands were cut into small pieces, following 10x dilution in ice-cold PBS (portion of the salivary gland intended for the determination of carbonyl groups were 10x diluted in 50 mM phosphate buffer), and homogenized with the addition of the protease inhibitor (1 tablet/10 mL of the buffer) (Complete Mini Roche, France) on ice with a glass homogenizer (Omni TH, Omni International, Kennesaw, GA, USA). Next, homogenates were sonificated with an ultrasonic cell disrupter (1800 J per sample, 20 s × 3, on ice) (UP400S, Hielscher, Teltow, Germany). The homogenates were spun (10 min, 4°C, 5000 g, MPW 351, MPW Med. Instruments, Warsaw, Poland). The resulting supernatants were analysed on the same day.

### 2.3. Assays

The plasma insulin and free fatty acids (FFA), plasma and salivary glands PC, 4-HNE protein adduct, oxy-LDL/MDA, 8-isoP, and 8-OHdG, total protein, TAS, TOS, and OSI were determined. Data were shown as the total amount (ratio of the examined parameter to total protein). Normalization to total proteins made it possible to assess differences in the ratio of biochemical analytes present in the biological fluids and tissue homogenates. All determinations were performed in duplicate. The final result is the arithmetic mean of the two measurements.

Plasma FFA concentrations were determined using the method described by Bligh and Dyer [18]. The plasma pH was measured using a pH meter SevenMulti, Mettler Toledo.

The insulin, 4-HNE protein adduct, oxy-LDL/MDA, 8-isoP, and 8-OHdG were determined by ELISA using commercially available kits (Shibayagi Co., Gunma, Japan; Cell Biolabs, Inc. San Diego, CA, USA; Immundiagnostik, Bensheim, Germany; Cayman Chemicals, Ann Arbor, MI, USA; and USCN Life Science, Wuhan, China, resp.) following the attached instructions. The supernatants, plasma, controls, and standards were incubated in microplate wells coated with monoclonal antibody to insulin, 4-HNE protein adduct, oxy-LDL/MDA, 8-isoP, and 8-OHdG. Next, avidin conjugated to horseradish peroxidase (HRP) was added and incubated, followed by incubations with the TMB substrate. Only plasma or supernatants that contained complex insulin, 4-HNE protein adduct, oxy-LDL/MDA, 8-isoP or 8-OHdG, biotin-conjugated antibody, and enzyme-conjugated avidin changed their colour. The reaction was terminated by the addition of sulphuric acid and the colour change was determined spectrophotometrically (microplate reader, Mindray MR-96, China).

The PC was determined according to the method introduced by Reznick and Packer [19]. The supernatant and plasma were incubated with 10 mM DNPH (2,4-dinitrophenylhydrazine; POCH S.A. (Polskie Odczynniki Chemiczne Spółka Akcyjna, Gliwice, Poland)) in 2.5 M HCl. The PC was calculated from the peak (355–390 nm) absorbance using the molar absorption coefficient *ε* = 22,000 M^−1^cm^−1^. Guanidine hydrochloride (Sigma Chemical Co., St. Louis, MO, USA) was used as a blank.

The TAS was assessed with a kit supplied by Randox (Crumlin, UK). ABTS (2,2′azino-di-[3-ethylbenzthiazoline sulphonate]) was incubated with peroxidase (metmyoglobin) and hydrogen peroxide to yield the cation ABTS^+^. ABTS^+^ had a blue-green colour, which was assessed spectrophotometrically at the wavelength 600 nm (microplate reader, Mindray MR-96, China). Antioxidants present in the sample reduce this colour formation proportional to their content.

The TOS determination was performed by commercial kit (PerOx, TOS/TOC) supplied by Immune Diagnostic (Bensheim, Germany). In this assay, peroxidase reacts with peroxides in the sample followed by the conversion of TMB to a coloured product. Addition of the stop solution stopped the reaction and caused change in colour. Absorbance of the sample was measured at 450 nm in a microtiter plate reader (Microplate Reader, Mindray MR-96, China). The quantification was performed by the delivered calibrator.

The oxidative stress index was calculated from the formula TOS/TAS × 100 [20].

The protein concentration was assessed by the bicinchoninic acid method (BCA), with bovine serum albumin as a standard (Thermo Scientific PIERCE BCA Protein Assay Kit, Rockford, IL, USA).

### 2.4. Statistical Analysis

Statistical analysis was performed using Statistica version 10.0 (Statsoft, Cracow, Poland). To show the significant differences between groups, the Kruskal-Wallis ANOVA test was performed. The Spearman Correlation Coefficient was used to study the associations between the variables. Results were presented as a median and minimum and maximum. The statistical significance was defined as *p* ≤ 0.05.

## 3. Results

### 3.1. Effect of Streptozotocin-Induced Diabetes on Body Weight, Plasma Insulin and pH, Fatty Acids, Glucose Concentration, Salivary Glands Weight, and Food Intake

Streptozotocin-induced diabetes caused reduction in the body weight in the DM groups as compared to the control groups in the first (12%, *p* = 0.03) and second (19.5%, *p* = 0.042) week of the experiment. In the control group, the body weight of rats was significantly increased between the first and the second week of the study (7%, *p* = 0.026), whereas the diabetic rats weight remained the same during the study (Table 1). Furthermore, in both periods of streptozotocin-induced diabetes a dramatic reduction in fasting plasma insulin concentration was observed (below the lower limit of detection), followed by 2.5 (*p* = 0.001) and 5.6 times (*p* = 0.0001), respectively, elevation in glucose concentration in DM groups as compared to the control rats. In the control group the glucose concentration remained the same during the study, whereas the diabetic rats showed significantly increased blood glucose concentrations between the first and the second week of the study (131%, *p* = 0.003) (Table 1). The plasma FFA concentration increased by 60%, *p* = 0.001 (1st week), and 78%, *p* = 0.001 (2nd week), in DM in comparison to the control group. In the control and DM groups, the FFA blood concentration remained the same during the study (Table 1). The plasma pH was similar in all groups during the experiment (Table 1). The weight salivary glands were similar in both DM and control groups in the first and the second week of the experiment. In the control and the DM groups, the salivary glands weight remained the same during the study (Table 2).

### 3.2. Effect of Streptozotocin-Induced Diabetes on Salivary Unstimulated and Stimulated Flow Rate

The median of the unstimulated flow rate was significantly 15% (*p* = 0.034) reduced in the DM rats as compared to the control rats only in the second week of the study. The median of the stimulated flow rate was significantly reduced in the DM rats as compared to the control rats throughout the duration of streptozotocin-induced diabetes (28%, *p* = 0.03, and 39%, *p* = 0.011, resp.) (Table 1).

### 3.3. Effect of Streptozotocin-Induced Diabetes on Plasma TAS, TOS, OSI, PC, 4-HNE Protein Adduct, oxy-LDL/MDA, 8-isoP, and 8-OHdG

One- and two-week lasting streptozotocin-induced diabetes resulted in a significantly higher median of the total amount of TOS (*p* = 0.0001 and *p* = 0.0002, resp.), OSI (*p* = 0.003 and *p* = 0.001, resp.), PC (*p* = 0.046 and *p* = 0.049, resp.), 4-HNE protein adduct (*p* = 0.00001 and *p* = 0.00001, resp.), oxy-LDL/MDA (*p* = 0.009 and *p* = 0.0006, resp.), and 8-isoP (*p* = 0.01 and *p* = 0.007, resp.) in plasma of the DM rats as compared to the control rats. The median of the total amount of plasma TAS of DM rats was significantly lower (*p* = 0.003) as compared to the median of the total amount of plasma TAS of the control rats, whereas the median of the total amount of 8-OHdG (*p* = 0.009) in plasma of DM rats was significantly elevated in the second week of the study as compared to the control rats (Table 3).

### 3.4. Effect of Streptozotocin-Induced Diabetes on Parotid Glands TAS, TOS, OSI, PC, 4-HNE Protein Adduct, oxy-LDL/MDA, 8-isoP, and 8-OHdG

**Figure 1:**
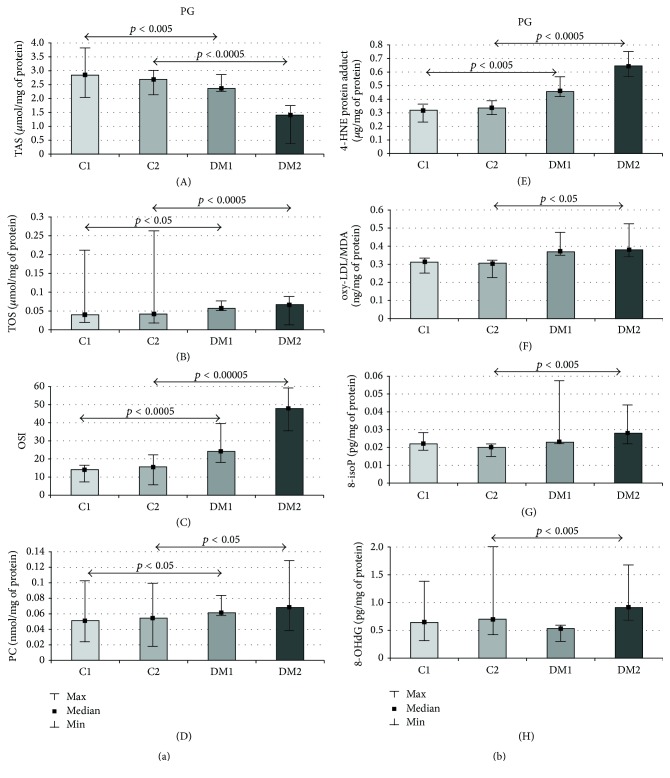
(a) Parotid gland TAS, TOS, OSI, and PC of the control and streptozotocin-diabetic rats. PG: parotid gland, C1: control group in the first week of the experiment, C2: control group in the second week of the experiment, DM1: diabetic group in the first week of the experiment, DM2: diabetic group in the second of the experiment, TAS: total antioxidant status, TOS: total oxidant status, OSI: oxidative status index, PC: protein carbonyls, *p*: statistical significance < 0.05, min: minimum, and max: maximum. (b) Parotid gland 4-HNE protein adduct, oxy-LDL/MDA, 8-isoP, and 8-OHdG of the control and streptozotocin-diabetic rats. PG: parotid gland, C1: control group in the first week of the experiment, C2: control group in the second week of the experiment, DM1: diabetic group in the first week of the experiment, DM2: diabetic group in the second of the experiment, 4-HNE protein adduct: 4-hydroxynonenal protein adduct, oxy-LDL/MDA: oxidized and/or MDA-modified LDL-cholesterol, 8-isoP: 8-isoprostanes, 8-OHdG: 8-hydroxy-D-guanosine, *p*: statistical significance < 0.05, M: median, min: minimum, and max: maximum.

The medians of the total amount of TAS (17%, *p* = 0.002, and 48%, *p* = 0.0001, resp.) were significantly reduced, whereas the medians of the total TOS (43%, *p* = 0.006, and 60%, *p* = 0.0001, resp.), OSI (72%, *p* = 0.0001, and 207%, *p* = 0.00001, resp.), PC (20%, *p* = 0.03, and 25%, *p* = 0.01, resp.) (Figure 1(a)), and 4-HNE protein adduct (43%, *p* = 0.001, and 92%, *p* = 0.0001, resp.) (Figure 1(b)) were significantly elevated in the parotid glands of the DM rats as compared to the control rats at each step of the experiment. The medians of the total oxy-LDL/MDA (24%, *p* = 0.03), 8-isoP (40%, *p* = 0.004), and 8-OHdG (30%, *p* = 0.001) were significantly higher in the parotid glands of the DM rats as compared to the control rats only in the second week of the streptozotocin-induced diabetes (Figure 1(b)).

### 3.5. Effect of Streptozotocin-Induced Diabetes on Submandibular Glands TAS, TOS, OSI, PC, 4-HNE Protein Adduct, oxy-LDL/MDA, 8-isoP, and 8-OHdG

**Figure 2:**
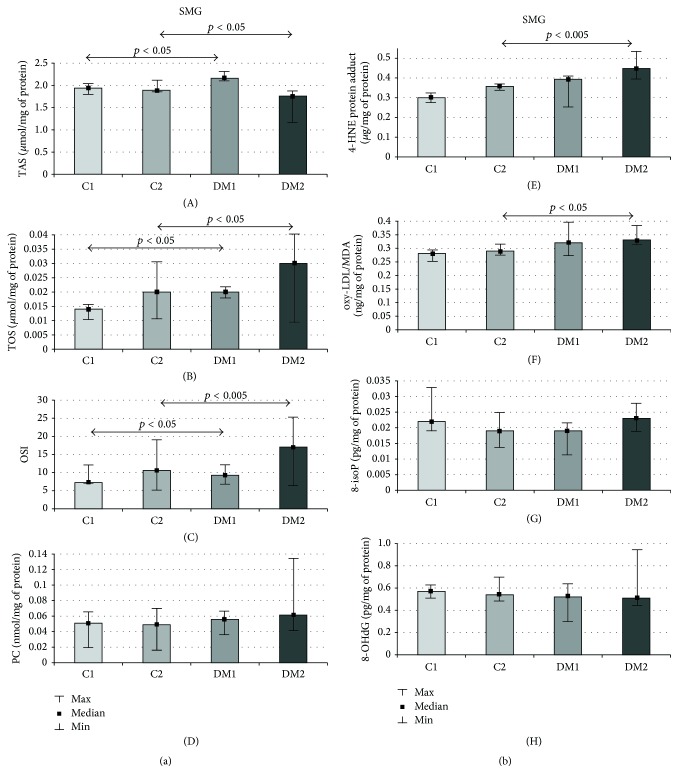
(a) Submandibular gland TAS, TOS, OSI, and PC of the control and streptozotocin-diabetic rats. SMG: submandibular gland, C1: control group in the first week of the experiment, C2: control group in the second week of the experiment, DM1: diabetic group in the first week of the experiment, DM2: diabetic group in the second of the experiment, TAS: total antioxidant status, TOS: total oxidant status, OSI: oxidative status index, PC: protein carbonyls, *p*: statistical significance < 0.05, min: minimum, and max: maximum. (b) Submandibular gland 4-HNE protein adduct, oxy-LDL/MDA, 8-isoP, and 8-OHdG of the control and streptozotocin-diabetic rats. SMG: submandibular gland, C1: control group in the first week of the experiment, C2: control group in the second week of the experiment, DM1: diabetic group in the first week of the experiment, DM2: diabetic group in the second of the experiment, 4-HNE protein adduct: 4-hydroxynonenal protein adduct, oxy-LDL/MDA: oxidized and/or MDA-modified LDL-cholesterol, 8-isoP: 8-isoprostanes, 8-OHdG: 8-hydroxy-D-guanosine, *p*: statistical significance < 0.05, M: median, min: minimum, and max maximum.

The medians of TAS were significantly higher (11%, *p* = 0.04) in diabetic submandibular glands as compared to the healthy control after one week of streptozotocin-induced diabetes, whereas the medians of the total amount of TOS (43%, *p* = 0.03, and 50%, *p* = 0.02, resp.) and OSI (28%, *p* = 0.03, and 61%, *p* = 0.002, resp.) were significantly upregulated in the submandibular glands of DM rats as compared to the control rats throughout the duration of streptozotocin-induced diabetes (Figure 2(a)). The median of the total TAS was significantly reduced (7%, *p* = 0.04) (Figure 2(a)), whereas the medians of the total amount of oxy-LDL/MDA (14%, *p* = 0.02) and 4-HNE protein adduct (25%, *p* = 0.002) were significantly elevated in the submandibular glands of the DM rats as compared to the control rats only in the second week of the streptozotocin-induced diabetes (Figure 2(b)).

#### 3.5.1. Parotid versus Submandibular Glands

*Diabetes*. Parotid glands of the diabetic rats showed significantly higher medians of the total amount of TAS (10%, *p* = 0.039), TOS (185%, *p* = 0.0002), OSI (161%, *p* = 0.0001), PC (10%, *p* = 0.02), 4-HNE protein adduct (15%, *p* = 0.032), oxy-LDL/MDA (15%, *p* = 0.03), and 8-isoP (21%, *p* = 0.04) in the first week of the streptozotocin-induced diabetes as compared to the submandibular glands of DM rats.

However, in the second week of the study parotid glands of DM rats showed no significant differences only for TAS; all other parameters, TOS (123%, *p* = 0.002), OSI (181%, *p* = 0.0001), PC (11%, *p* = 0.04), 4-HNE protein adduct (44%, *p* = 0.001), oxy-LDL/MDA (14%, *p* = 0.03), 8-isoP (21%, *p* = 0.039) and 8-OHdG (78%, *p* = 0.001), were significantly higher as compared to the submandibular gland of diabetic rats (Table 2).

*Control Rats*. Parotid glands of the control rats showed higher medians of total amounts of TAS (46%, *p* = 0.001; 42%, *p* = 0.001, resp.) and TOS (185%, *p* = 0.0001; 110%, *p* = 0.001) as compared to the submandibular glands of the control rats, both the first and the second week of the experiment (Table 2).

*Correlation*

*One Week*

*Submandibular Glands*. Correlation was observed between the total amount of TAS and 4-HNE protein adduct and 8-isoP (*p* = 0.035, *r* = −0.45; *p* = 0.043, *r* = −0.37, resp.)

*2 Week*

*Submandibular Glands*. Correlation was observed between glucose concentrations and the total amount of 4-HNE protein adduct and oxy-LDL/MDA (*p* = 0.033, *r* = 0.37; *p* = 0.011, *r* = 0.57, resp.)

*Parotid Glands*. Correlation between the total amount of 4-HNE protein adduct and SWS (*p* = 0.017, *r* = −0.61) and correlation between glucose concentrations and a total amount of 4-HNE protein adduct, oxy-LDL/MDA, PC, and 8-isoP (*p* = 0.023, *r* = 0.57; *p* = 0.009, *r* = 0.71; *p* = 0.034, *r* = 0.54; *p* = 0.01, *r* = 0.64, resp.) were observed.

## 4. Discussion

In this paper, we assessed the level and progression of the oxidative damage to the diabetic salivary glands using various biomarkers of oxidative stress and oxidative injury. We investigated the relationship between oxidative stress and secretory function of the salivary glands in the course of streptozotocin-induced diabetes.

The present model of type 1 diabetes was based on streptozotocin- (STZ-) induced diabetes. The dose of STZ is a determining factor for the extent of its diabetogenic action. Experimental diabetes type 1 is induced in animals using STZ in doses from 45 to 100 mg/dL [21]: doses of STZ < 40 mg/dL may not induce diabetes and doses higher than 80 mg/dL may lead to excess rats mortality [21]. Based on the literature [22, 23] and our own experience, we chose a dose of 50 mg/dL, because this amount determines sufficient diabetogenic effect at 100% survival of rats over two time periods [12]. STZ model uses the fact that STZ is selectively toxic to beta (*β*) cells of the pancreas [24] and is recommended as a clinically relevant animal model and it is most commonly used to explore human diabetes type 1 [21]. In line with our expectations, we received severe hyperglycaemia with simultaneous reduction of fasting plasma insulin levels (below the limit of detection) as early as 1 week after STZ injection. The obtained results demonstrated the destruction of pancreatic *β* cells and, consequently, endocrine failure of this organ. We also experienced progression of the impaired endocrine function of pancreatic *β* cells; between the first and the second week of the study, diabetic rats showed significantly increased blood glucose concentrations. Furthermore, despite the fact that diabetic rats consumed similar amounts of food as the control rats, their body weight was significantly lower as compared to the control group.

Under physiological conditions, there is a balance between the generation and elimination of ROS in living organisms, and therefore oxidative damage cannot be observed. However, when this redox balance is disrupted, an elevated production of ROS is observed, followed by an increase in ROS modified molecules, which are widely used biomarkers of oxidative damage. It is considered that the assessment of oxidative injury using only single oxidative modification marker is not sufficient in organisms due to “the different sensitivity, dynamics, nature, and results of ROS-involving process” [25].

Several products derived from biomolecule oxidation were detected in various diabetic organs and plasma [26–30]; however, the only examined marker of oxidative stress in diabetic salivary glands is MDA. Lushchack [25] claims that the reaction of MDA with thiobarbituric acid is not specific and many compounds (amino acids, carbohydrates, and aldehydes) may react with TBA and may interfere with the assay, so these methods as well as interpretation of the obtained results should be evaluated with caution. On one hand, Cebe at al. [9] recently proposed that the pretreatment of biological samples for avoiding the possible interferences derived other reactive aldehydes which could be eliminated according to their recent sample preparation method. In the present experiment, we decided to evaluate the recommended and most commonly used oxidative injury biomarkers: PC, 8-isoP, oxy-LDL/MDA, 4-HNE protein adduct, and 8-OHdG as well as OSI which is an objective assessment of the relationship between total antioxidant mechanisms and the oxidants level [20].

Oxidative damage in the form of a significant increase in 4-HNE protein adduct and PC in the first week after the STZ administration was noted only in the salivary parotid glands. However, a significant increase in the total amount of OSI in the diabetic submandibular glands as compared to the control demonstrates the redox imbalance also in this gland. The submandibular glands, in contrast to the diabetic parotid glands, seem to prevent their destruction in the process of OS through antioxidant defence system activation (TAS). The negative correlation between the total amounts of TAS, 4-HNE protein adduct, and 8-isoP shows that the increase in the antioxidant capacity elevates cell resistance to oxidative damage and it is sufficient to maintain ROS modified biomolecules within the basal state [31]. It is difficult to explain these differences based solely on the results of the present experiment. Increased TAS levels in the submandibular glands may suggest, on the one side, the adaptive response to the excess production of ROS in this gland; however, on the other hand, it may be an attempt to compensate dysfunctional parotid glands in terms of antioxidant features. These changes may also suggest that, at least in the early stages of type 1 diabetes, the oral cavity will be sufficiently protected against free radicals, even if parotid glands, the physiological source of oral antioxidants, are deficient.

ROS formation in diabetes is directly associated with hyperglycaemia. Hyperglycaemia promotes the generation of free radicals by modulation of the mitochondrial respiratory chain, nonenzymatic glucose autoxidation, and protein glycation [32] and a reduction in the antioxidant capacity [33, 34]. The present results showed that oxidative injury in both glands is directly influenced by hyperglycaemia only in the second week of the experiment, which was observed by a positive correlation between plasma glucose levels and a total amount of 4-HNE protein adduct and oxy-LDL/MDA, for both glands, and 8-isoP and PC for the parotid gland. It is not surprising that the progression of endocrine insufficiency of the pancreas was accompanied by an extensive oxidation of major cellular components in both salivary glands, in addition to a significant reduction in TAS. Greater intensification of oxidative damage was observed in the diabetic parotid salivary glands. However, in the diabetic parotid gland, in addition to the 4-HNE protein adduct and PC and also oxy-LDL/MDA, 8-isoP and 8-OHdG were significantly higher as compared to the control glands. Selectively, from all evaluated oxidation products, a significant increase in the lipid peroxidation products such as 4-HNE protein adduct and oxy-LDL/MDA in the diabetic submandibular glands versus control could be a proof that these diabetic glands undergo an early stage of oxidative damage. Lipid peroxidation is thought to be the earliest marker of oxidative damage occurring via OS, which results from the fact that cell membrane is first exposed to free radicals before the other cellular components undergo oxidative modification [35]. It should be noted that in the present study we investigated only selected damage products. Determination of the other markers of oxidative damage (e.g., advanced oxidation protein product, disulphide groups, and others) may partially contradict this conclusion. Obviously, we cannot exclude that hyperglycaemia, increased endothelial permeability, allows passage of oxidation products from vessels to salivary glands nor that evaluated oxidation products arise directly or only in the salivary glands, especially when we observed an increase in overall plasma oxidative modification and also cellular redox imbalance already in the first week after the STZ administration. However, the lack of any correlation between plasma and salivary oxidative stress parameters suggests that observed results may be caused by processes taking place in the salivary glands, independently from plasma/general oxidative stress.

Interestingly, regardless of the oxidative stress and oxidative damage intensity, we observed that the capacity of the parotid glands in the response to external stimuli (SWS) is depressed throughout the whole experiment. Basal activity (UWS) of the submandibular glands versus control is depressed only in the second week of the experiment; in the first week of the study, despite the shifting of the antioxidants/oxidants balance towards the oxidative status (↑OSI), the mechanisms of saliva secretion in the diabetic submandibular glands are unaffected, which is reflected as no significant differences in the UWS as compared to healthy control. It should be noted that, in the absence of stimulation, the submandibular gland provides about 60% of total salivary secretion and it is the main source of unstimulated saliva. After saliva stimulation, only the parotid glands increase their secretion by about 10–15%, and thus parotid glands may be considered to be the main source of stimulated saliva [36].

The observed negative correlation between 4-HNE protein adduct and a stimulated salivary flow in the second week seems interesting due to the fact that the 4-HNE protein adduct is able to enhance the expression of proinflammatory cytokines and metalloproteinase production in the course of mitochondrial ROS-mediated stimulation of Akt/NF-kappaB signalling pathways [37]. It has been shown that inflammatory mediators and matrix metalloproteinase may decrease the response of residual acinar cells to acetylcholine and/or block their receptors leading to a reduction in saliva production and secretion [38].

Our results showed greater extent and diversity of oxidative injury in the parotid diabetic glands than in the submandibular diabetic glands, which we also observed in morbid obesity [39]. Perhaps the observed differences may result from differing intensity of morphological changes in both diabetic salivary glands [11, 40, 41]. Histological analysis of dietary salivary glands revealed, amongst other things, a massive accumulation of adipocytes in the parenchyma of vesicular and mucosal cells, which can be seen particularly in parotid glands and slightly marked in submandibular glands. By releasing monocyte chemoattractant protein-1 (MCP-1) the adipocytes cause an influx of monocytes and promote their transformation into macrophages [42]. Moreover, synthesis of MCP-1 is induced by the 4-HNE protein adduct [43]. Macrophages release cytokines (TNF, IL6, and IL1*β*) and promote the development of inflammation which leads to the stimulation of respiratory processes in the phagocytic cells, activation of NADPH oxidase, and formation of large amounts of ROS. Perhaps, these additional sources of free radicals exceed the antioxidant/repair capabilities of the parotid glands and their oxidative damage is also observed throughout the whole experiment.

Analyzing the results of our experiment, attention should be paid to its limitations. The present research has been conducted on an animal experimental model; although such model provides invaluable assistance, it is not entitled to its direct impact on humans. The dose of STZ, duration of STZ diabetes, and determination of the other markers of oxidative damage could partially contradict our results.

## 5. Conclusion

1. Both parotid and submandibular glands undergo oxidative stress in the course of streptozotocin diabetes, irrespective of the duration of the disease.
2. The parotid glands seem more exposed and vulnerable than submandibular glands to an oxidant attack generated in the course of streptozotocin diabetes, regardless of the duration of the disease.
3. Oxidative damage in the course of streptozotocin diabetes caused the dysfunction of the salivary glands, wherein only the reduction of the stimulated saliva secretion is observed in the first week of the experiment. In the advanced stages of the disease, simulated saliva secretion is significantly more reduced than the secretion of nonstimulated saliva.
4. Oxidative damage to the salivary glands in the course of STZ diabetes seems to be caused by processes taking place in the salivary glands, independently from plasma/general oxidative stress.

## Figures and Tables

**Table 1:** The body weight, salivary unstimulated and stimulated flow rate, and the concentrations of plasma: insulin, glucose, free fatty acids, and pH of the control and streptozotocin-diabetic rats.

	Body weight (mg) M (min–max)	Salivary flow rate (*μ*L/min) M (min–max)		Insulin (ng/mL) M (min–max)	Glucose (mg/dL) M (min–max)	FFA (nmol/mL) M (min–max)	pH M (min–max)
		UWS	SWS				
C1	262.5 (223−279)	0.35 (0.2−0.56)	88.6 (75.9−130.2)	0.854 (0.799−1.456)	95.25 (76.53−99.74)	97.77 (74.39−103.87)	7.71 (7.7−7.9)
C2	282.5 (264−297)	0.36 (0.22−0.61)	86.4 (70.2−109.8)	1.066 (0.991−1.682)	97.86 (80.26−102.47)	91.56 (88.77−96.72)	7.71 (7.6−7.94)
DM1	230.5 (201−260)	0.32 (0.06−0.52)	63.79 (59.94−78.7)	0.0	237 (129−255)	156.43 (133.65−198.66)	7.71 (7.49−7.9)
DM2	227.5 (209−250)	0.27 (0.067−0.91)	52.7 (48.56−69.24)	0.0	548.02 (425.38−594.33)	162.98 (143.52−186.52)	7.71 (7.68−7.93)
*p* (C1 : C2)	<0.05 (↑)	ns	ns	ns	ns	ns	ns
*p* (DM1 : DM2)	ns	ns	ns	ns	<0.005 (↑)	ns	ns
*p* (C1 : DM1)	<0.05 (↓)	ns	<0.05 (↓)	<0.000005 (↓)	<0.005 (↑)	<0.005 (↑)	ns
*p* (C2 : DM2)	<0.05 (↓)	<0.05 (↓)	<0.05 (↓)	<0.000005 (↓)	<0.0005 (↑)	<0.005 (↑)	ns

C1: control group in the first week of the experiment, C2: control group in the second week of the experiment, DM1: diabetic group in the first week of the experiment, DM2: diabetic group in the first second of the experiment, SWS: stimulated whole saliva, UWS: unstimulated whole saliva, insulin: plasma insulin concentration, glucose: *plasma* glucose concentration, FFA: *plasma* free fatty acids concentration, M: median, min: minimum, max: maximum, ns: not significant, *p*: statistical significance < 0.05, (↓): decrease, and (↑): increase.

**Table 2:** Comparison of the examined parameters between parotid and submandibular glands of the control and streptozotocin-diabetic rats.

Group *N* = 8	PG	SMG	*p* PG : SMG
Salivary glands weight (g) M (min–max)			
C1	0.09 (0.08–0.12)	0.19 (0.18–0.22)	ns
C2	0.09 (0.79–0.13)	0.21 (0.17–0.24)	ns
DM1	0.09 (0.07–0.09)	0.21 (0.14–0.24)	ns
DM2	0.07 (0.06–0.09)	0.18 (0.11–0.19)	ns

TAS (*μ*mol/mg of protein) M (min–max)			
C1	2.84 (2.01–3.81)	1.94 (1.75–2.06)	<0.005 (↑)
C2	2.69 (2.15–2.99)	1.89 (1.84–2.10)	<0.005 (↑)
DM1	2.36 (2.30–2.89)	2.16 (2.10–2.30)	<0.05 (↑)
DM2	1.40 (0.42–1.74)	1.76 (1.17–1.82)	ns

TOS (*μ*mol/mg of protein) M (min–max)			
C1	0.04 (0.02–0.21)	0.014 (0.01–0.11)	<0.0005 (↑)
C2	0.042 (0.02–0.26)	0.02 (0.01–0.30)	<0.005 (↑)
DM1	0.057 (0.05–0.08)	0.02 (0.018–0.021)	<0.0005 (↑)
DM2	0.067 (0.01–0.09)	0.03 (0.008–0.04)	<0.005 (↑)

OSI M (min–max)			
C1	14.08 (6.32–17.52)	7.22 (7.17–12.17)	ns
C2	15.61 (5.21–22.5)	10.58 (4.97–19.35)	ns
DM1	24.15 (19.97–39.81)	9.26 (6.67–12.54)	<0.0005 (↑)
DM2	47.86 (36.52–59.86)	17.05 (6.02–25.33)	<0.0005 (↑)

PC (nmol/mg of protein) M (min–max)			
C1	0.051 (0.025–0.10)	0.051 (0.019–0.065)	ns
C2	0.055 (0.019–0.100)	0.049 (0.016–0.070)	ns
DM1	0.061 (0.058–0.084)	0.056 (0.036–0.064)	<0.05 (↑)
DM2	0.068 (0.039–0.127)	0.061 (0.040–0.136)	<0.05 (↑)

4-HNE protein adduct (*μ*g/mg of protein) M (min–max)			
C1	0.32 (0.24–0.38)	0.30 (0.28–0.34)	ns
C2	0.34 (0.30–0.39)	0.36 (0.33–0.37)	ns
DM1	0.46 (0.43–0.57)	0.39 (0.26–0.41)	<0.05 (↑)
DM2	0.65 (0.57–0.74)	0.45 (0.39–0.54)	<0.005 (↑)

oxy-LDL/MDA (ng/mg of protein) M (min–max)			
C1	0.31 (0.26–0.33)	0.28 (0.25–0.29)	ns
C2	0.31 (0.22–0.32)	0.29 (0.27–0.32)	ns
DM1	0.37 (0.35–0.49)	0.32 (0.28–0.4)	<0.05 (↑)
DM2	0.38 (0.35–0.53)	0.33 (0.32–0.38)	<0.05 (↑)

8-isoP (pg/mg of protein) M (min–max)			
C1	0.022 (0.019–0.029)	0.022 (0.019–0.033)	ns
C2	0.020 (0.015–0.021)	0.019 (0.014–0.025)	ns
DM1	0.023 (0.021–0.057)	0.019 (0.011–0.021)	<0.05 (↑)
DM2	0.028 (0.021–0.044)	0.023 (0.019–0.028)	<0.05 (↑)

8-OHdG (pg/mg of protein) M (min–max)			
C1	0.64 (0.30–1.40)	0.57 (0.51–0.61)	ns
C2	0.70 (0.41–2.00)	0.54 (0.49–0.68)	ns
DM1	0.53 (0.31–0.63)	0.52 (0.32–0.63)	ns
DM2	0.91 (0.74–1.65)	0.51 (0.41–0.97)	<0.005 (↑)

C1: control group in the first week of the experiment, C2: control group in the second week of the experiment, DM1: diabetic group in the first week of the experiment, DM2: diabetic group in the first second of the experiment, PG: parotid gland, SMG: submandibular gland, TAS: total antioxidant status, TOS: total oxidant status, OSI: oxidative status index, PC: protein carbonyls, 4-HNE protein adduct: 4-hydroxynonenal protein adduct, oxy-LDL/MDA: oxidized and/or MDA-modified LDL-cholesterol, 8-isoP: 8-isoprostanes, 8-OHdG: 8-hydroxy-D-guanosine, ns: not significant, *p*: statistical significance < 0.05, M: median, min: minimum, max: maximum, and (↑): increase.

**Table 3:** Plasma TAS, TOS, OSI, protein carbonyls (PC), 4-HNE protein adduct, oxy-LDL/MDA, 8-isoprostanes (8-isoP), and 8-OHdG of the control and streptozotocin-diabetic rats.

	TAS (*μ*mol/mg of protein) M (min–max)	TOS (*μ*mol/mg of protein) M (min–max)	OSI M (min–max)	PC (pmol/mg of protein) M (min–max)	4-HNE protein adduct (ng/mg of protein) M (min–max)	oxy-LDL/MDA (pg/mg of protein) M (min–max)	8-isoP (pg/mg of protein) M (min–max)	8-OHdG (pg/mg of protein) M (min–max)
C1	0.1 (0.06–0.15)	1.03 (0.32–2.44)	1.03 (0.095–1.17)	1.41 (1.07–1.48)	0.004 (0.001–0.005)	1.57 (0.99–2.64)	0.0019 (0.0018–0.0022)	0.20 (0.17–0.24)
C2	0.1 (0.05–0.16)	1.05 (0.88–1.28)	0.7 (0.47–1.13)	1.48 (1.31–2.01)	0.005 (0.001–0.007)	1.62 (1.47–2.24)	0.0018 (0.0011–0.0023)	0.21 (0.19–0.22)
DM1	0.16 (0.09–0.20)	2.84 (2.54–3.27)	1.78 (1.57–2.51)	1.57 (1.51–2.11)	6.8 (6.7–7.5)	2.29 (1.21–2.95)	0.0023 (0.0019–0.0026)	0.26 (0.24–0.30)
DM2	0.077 (0.05–0.15)	2.67 (2.48–2.74)	1.31 (1.21–1.83)	1.59 (1.09–2.19)	6.5 (6.0–7.7)	3.65 (3.08–4.01)	0.0022 (0.0017–0.0026)	0.26 (0.25–0.29)
*p* (C1 : C2)	ns	ns	ns	ns	ns	ns	ns	ns
*p* (DM1 : DM2)	ns	ns	ns	ns	ns	<0.005 (↑)	ns	ns
*p* (C1 : DM1)	ns	<0.0005 (↑)	<0.005 (↑)	<0.05 (↑)	<0.00005 (↑)	<0.05 (↑)	<0.05 (↑)	ns
*p* (C2 : DM2)	<0.05 (↓)	<0.0005 (↑)	<0.005 (↑)	<0.05 (↑)	<0.00005 (↑)	<0.005 (↑)	<0.05 (↑)	<0.05 (↑)

C1: control group in the first week of the experiment, C2: control group in the second week of the experiment, DM1: diabetic group in the first week of the experiment, DM2: diabetic group in the first second of the experiment, TAS: total antioxidant status, TOS: total oxidant status, OSI: oxidative status index, PC: protein carbonyls, 4-HNE protein adduct: 4-hydroxynonenal protein adduct, oxy-LDL/MDA: oxidized and/or MDA-modified LDL-cholesterol, 8-isoP: 8-isoprostanes, 8-OHdG: 8-hydroxy-D-guanosine, ns: not significant, *p*: statistical significance < 0.05, M: median, min: minimum, max: maximum, (↓): decrease, and (↑): increase.

## References

[1] Knaś M., Karaszewska K., Szajda S. D., Zarzycki W., Dudzik D., Zwierz K. (2006). Saliva of patients with Type 1 diabetes: effect of smoking on activity of lysosomal exoglycosidases. *Oral Diseases*.

[2] Kurek K., Wiesiołek-Kurek P., Piotrowska D. M., Łukaszuk B., Chabowski A., Zendzian-Piotrowska M. (2014). Inhibition of ceramide de novo synthesis with myriocin affects lipid metabolism in the liver of rats with streptozotocin-induced type 1 diabetes. *BioMed Research International*.

[3] Bonnefont-Rousselot D., Bastard J. P., Jaudon M. C., Delattre J. (2000). Consequences of the diabetic status on the oxidant/antioxidant balance. *Diabetes and Metabolism*.

[4] Lushchak V. I. (2014). Classification of oxidative stress based on its intensity. *EXCLI Journal*.

[5] Çakatay U. (2005). Protein oxidation parameters in type 2 diabetic patients with good and poor glycaemic control. *Diabetes and Metabolism*.

[6] Grosicka-Maciąg E. (2011). Biological consequences of oxidative stress induced by pesticides. *Postpy Higieny i Medycyny Doświadczalnej*.

[7] Wang Z., Wang Y., Liu H., Che Y., Xu Y., E L. (2015). Age-related variations of protein carbonyls in human saliva and plasma: is saliva protein carbonyls an alternative biomarker of aging?. *AGE*.

[8] Lovell M. A., Soman S., Bradley M. A. (2011). Oxidatively modified nucleic acids in preclinical Alzheimer's disease (PCAD) brain. *Mechanisms of Ageing and Development*.

[9] Cebe T., Atukeren P., Yanar K. (2014). Oxidation scrutiny in persuaded aging and chronological aging at systemic redox homeostasis level. *Experimental Gerontology*.

[10] Nogueira F. N., Carvalho A. M., Yamaguti P. M., Nicolau J. (2005). Antioxidant parameters and lipid peroxidation in salivary glands of streptozotocin-induced diabetic rats. *Clinica Chimica Acta*.

[11] Deconte S. R., Da Silva Oliveira R. J., Calábria L. K. (2011). Alterations of antioxidant biomarkers and type I collagen deposition in the parotid gland of streptozotocin-induced diabetic rats. *Archives of Oral Biology*.

[12] Zalewska A., Knaś M., Maciejczyk M. (2015). Antioxidant profile, carbonyl and lipid oxidation markers in the parotid and submandibular glands of rats in different periods of streptozotocin induced diabetes. *Archives of Oral Biology*.

[13] Abuelo A., Hernández J., Benedito J. L., Castillo C. (2013). Oxidative stress index (OSi) as a new tool to assess redox status in dairy cattle during the transition period. *Animal*.

[14] Wohaieb S. A., Godin D. V. (1987). Alterations in free radical tissue-defense mechanisms in streptozocin-induced diabetes in rat. Effects of insulin treatment. *Diabetes*.

[15] Sabino-Silva R., Okamoto M. M., David-Silva A., Mori R. C., Freitas H. S., Machado U. F. (2013). Increased SGLT1 expression in salivary gland ductal cells correlates with hyposalivation in diabetic and hypertensive rats. *Diabetology and Metabolic Syndrome*.

[16] Picco D. C. R., Costa L. F., Delbem A. C. B., Sassaki K. T., Sumida D. H., Antoniali C. (2012). Spontaneously hypertensive rat as experimental model of salivary hypofunction. *Archives of Oral Biology*.

[17] Romero A. C., Ibuki F. K., Nogueira F. N. (2012). Sialic acid reduction in the saliva of streptozotocin induced diabetic rats. *Archives of Oral Biology*.

[18] Bligh E. G., Dyer W. J. (1959). A rapid method of total lipid extraction and purification. *Canadian Journal of Biochemistry and Physiology*.

[19] Reznick A. Z., Packer L. (1994). Oxidative damage to proteins: spectrophotometric method for carbonyl assay. *Methods in Enzymology*.

[20] Şen V., Uluca Ü., Ece A. (2014). Serum prolidase activity and oxidant-antioxidant status in children with chronic hepatitis B virus infection. *Italian Journal of Pediatrics*.

[21] Goyal S. N., Reddy N. M., Patil K. R. (2016). Challenges and issues with streptozotocin-induced diabetes-a clinically relevant animal model to understand the diabetes pathogenesis and evaluate therapeutics. *Chemico-Biological Interactions*.

[22] Szkudelski T. (2001). The mechanism of alloxan and streptozotocin action in B cells of the rat pancreas. *Physiological Research*.

[23] Mansour A. M., Abou Elkhier M. T., Ibrahim F. M. (2016). Effect of Aloe vera on submandibular salivary gland of streptozotocin-induced diabetic rats. *International Journal of Advanced Research*.

[24] Sakata N., Yoshimatsu G., Tsuchiya H., Egawa S., Unno M. (2012). Animal models of diabetes mellitus for islet transplantation. *Experimental Diabetes Research*.

[25] Lushchak V. I. (2014). Free radicals, reactive oxygen species, oxidative stress and its classification. *Chemico-Biological Interactions*.

[26] Broedbaek K., Weimann A., Stovgaard E. S., Poulsen H. E. (2011). Urinary 8-oxo-7,8-dihydro-2′-deoxyguanosine as a biomarker in type 2 diabetes. *Free Radical Biology and Medicine*.

[27] Dhaunsi G. S., Bitar M. S. (2004). Antioxidants attenuate diabetes-induced activation of peroxisomal functions in the rat kidney. *Journal of Biomedical Science*.

[28] Lee S. C., Chan J. C. N. (2015). Evidence for DNA damage as a biological link between diabetes and cancer. *Chinese Medical Journal*.

[29] Şakul A., Cumaoğlu A., Aydin E., Ari N., Dilsiz N., Karasu Ç. (2013). Age- and diabetes-induced regulation of oxidative protein modification in rat brain and peripheral tissues: Consequences of treatment with antioxidant pyridoindole. *Experimental Gerontology*.

[30] Kayali R., Telci A., Cakatay U. (2003). Oxidative protein damage parameters in plasma in chronic experimental diabetes in rats. *European Journal of Medical Research*.

[31] Cheeseman K. H., Burton G. W., Ingold K. U., Slater T. F. (1984). Lipid peroxidation and lipid antioxidants in normal and tumor cells. *Toxicologic Pathology*.

[32] Çakatay U., Kayali R. (2006). The evaluation of altered redox status in plasma and mitochondria of acute and chronic diabetic rats. *Clinical Biochemistry*.

[33] Kang J. H. (2003). Modification and inactivation of human Cu, Zn-superoxide dismutase by methylglyoxal. *Molecules and Cells*.

[34] Yuan Y., Jiao X., Lau W. B. (2010). Thioredoxin glycation: a novel posttranslational modification that inhibits its antioxidant and organ protective actions. *Free Radical Biology and Medicine*.

[35] Erel O. (2005). A new automated colorimetric method for measuring total oxidant status. *Clinical Biochemistry*.

[36] Dawes C. (2008). Salivary flow patterns and the health of hard and soft oral tissues. *Journal of the American Dental Association*.

[37] Lee S. J., Seo K. W., Yun M. R. (2008). 4-hydroxynonenal enhances MMP-2 production in vascular smooth muscle cells via mitochondrial ROS-mediated activation of the Akt/NF-*κ*B signaling pathways. *Free Radical Biology and Medicine*.

[38] Tzioufas A. G., Tsonis J., Moutsopoulos H. M. (2008). Neuroendocrine dysfunction in Sjögren's syndrome. *NeuroImmunoModulation*.

[39] Knaś M., Maciejczyk M., Sawicka K. (2015). Impact of morbid obesity and bariatric surgery on antioxidant/oxidant balance of the unstimulated and stimulated human saliva. *Journal of Oral Pathology & Medicine*.

[40] Mahay S., Adeghate E., Lindley M. Z., Rolph C. E., Singh J. (2004). Streptozotocin-induced type 1 diabetes mellitus alters the morphology, secretory function and acyl lipid contents in the isolated rat parotid salivary gland. *Molecular and Cellular Biochemistry*.

[41] Reuterving C.-O., Hägg E., Henriksson R., Holm J. (1987). Salivary glands in long-term alloxan-diabetic rats. A quantitative light and electron-microscopic study. *Acta Pathologica, Microbiologica, et Immunologica Scandinavica*.

[42] Solinas G., Karin M. (2010). JNK1 and IKK*β*: molecular links between obesity and metabolic dysfunction. *The FASEB Journal*.

[43] Leonarduzzi G., Chiarpotto E., Biasi F., Poli G. (2005). 4-hydroxynonenal and cholesterol oxidation products in atherosclerosis. *Molecular Nutrition and Food Research*.

